# Reduction of neutrophil activity decreases early microvascular injury after subarachnoid haemorrhage

**DOI:** 10.1186/1742-2094-8-103

**Published:** 2011-08-19

**Authors:** Victor Friedrich, Rowena Flores, Artur Muller, Weina Bi, Ellinor IB Peerschke, Fatima A Sehba

**Affiliations:** 1 Department of Neuroscience Mount Sinai School of Medicine, New York, NY 10029, USA; 2 Department of Neurosurgery Mount Sinai School of Medicine, New York, NY 10029, USA; 3 Department of Pathology Mount Sinai School of Medicine, New York, NY 10029, USA

## Abstract

**Background:**

Subarachnoid haemorrhage (SAH) elicits rapid pathological changes in the structure and function of parenchymal vessels (≤ 100 μm). The role of neutrophils in these changes has not been determined. This study investigates the role of neutrophils in early microvascular changes after SAH

**Method:**

Rats were either untreated, treated with vinblastine or anti-polymorphonuclear (PMN) serum, which depletes neutrophils, or treated with pyrrolidine dithiocarbamate (PDTC), which limits neutrophil activity. SAH was induced by endovascular perforation. Neutrophil infiltration and the integrity of vascular endothelium and basement membrane were assessed immunohistochemically. Vascular collagenase activity was assessed by *in situ *zymography.

**Results:**

Vinblastine and anti-PMN serum reduced post-SAH accumulation of neutrophils in cerebral vessels and in brain parenchyma. PDTC increased the neutrophil accumulation in cerebral vessels and decreased accumulation in brain parenchyma. In addition, each of the three agents decreased vascular collagenase activity and post-SAH loss of vascular endothelial and basement membrane immunostaining.

**Conclusions:**

Our results implicate neutrophils in early microvascular injury after SAH and indicate that treatments which reduce neutrophil activity can be beneficial in limiting microvascular injury and increasing survival after SAH.

## Background

Subarachnoid haemorrhage (SAH) is followed by pathological alterations in cerebral microvasculature (≤100 μm) [1-6]. These alterations develop rapidly (< 24 hours) and affect vascular structure and function. The structural alterations include corrugation and in some cases physical detachment of endothelium from the basal lamina, loss of endothelial antigens, accumulation of platelet aggregates in the vessel lumen, and degradation of collagen IV, the major protein of basal lamina [4,5,7,8]. Functional changes closely follow the structural alterations and include endothelial dysfunction, constriction, perfusion deficits, and permeability increases [4-7].

Previous studies have implicated luminal platelets in early microvascular pathology after SAH [5,6]. The contribution of platelets to microvascular injury may represent an inflammatory response to the rupture of the arterial wall, promoted by an initial reduction in cerebral blood flow. Neutrophils are another key component of the inflammatory cascade, and have the ability to generate pathologic changes in blood vessels. Overt activation of neutrophils is implicated in vessel wall pathology and in the progression of a variety of diseases and disorders including cardiovascular diseases, haemolytic uremic syndrome and stroke [9-12]. Marked neutrophil infiltration is also reported 3 days after SAH and is associated with an increased risk of developing vasospasm [13,14]. Recently, Provencio et al, [15,16] reported that prior depletion of circulating myeloid cells ameliorates SAH-induced reduction in the calibre of middle cerebral artery and, further, that neutrophils have accumulated in parietal lobe parenchyma at one day post-lesion. We have previously reported changes as early as 10 minutes post-haemorrhage in brain parenchymal microvessels, including platelet accumulations, increased microvascular collagenase activity, and destruction of microvascular basement membrane and blood-brain barrier [3,7,8]. We here address the possible role of neutrophils in the very early development of these microvascular pathologies. We report that pronounced neutrophil accumulation is present in brain microvessels and in brain parenchyma at 10 minutes post-haemorrhage. Furthermore inhibition of neutrophil-mediated effects by two different pharmacological strategies partially protected microvessels. These observations suggest that neutrophils may play a pivotal role in microvascular pathology following SAH and suggest neutrophils as potential targets in SAH therapies.

## Methods

All experimental procedures and protocols were approved by the Institutional Animal Care and Use Committee of the Mount Sinai Medical Center.

### Induction of subarachnoid haemorrhage

Male Sprague-Dawley rats (325-350 g) underwent experimental SAH using the endovascular suture model developed in this laboratory [17,18]. Briefly, rats were anesthetized with ketamine-xylazine (80 mg/kg+10 mg/kg; i.p.), transorally intubated, ventilated, and maintained on inspired isoflourane (1% to 2% in oxygen-supplemented room air). Rats were placed on a homeothermic blanket Harvard Apparatus, MA, USA) attached to a rectal temperature probe set to maintain body temperature at 37°C and positioned in a stereotactic frame. The femoral artery was exposed and cannulated for blood gas and blood pressure monitoring (ABL5, Radiometer America Inc. Ohio, USA). For measurement of intracranial pressure (ICP), the atlanto-occipital membrane was exposed and cannulated, and the cannula was affixed with methymethacrylate cement to a stainless steel screw implanted in the occipital bone. Cerebral blood flow (CBF) was measured by laser-Doppler flowmetry, using a 0.8 mm diameter needle probe (Vasamedics, Inc., St. Paul, MN, USA) placed over the skull away from large pial vessels in the distribution of the middle cerebral artery.

SAH was induced by advancing a suture retrogradely through the ligated right external carotid artery (ECA), and distally through the internal carotid artery (ICA) until the suture perforated the intracranial bifurcation of the ICA. This event was detected by a rapid rise in ICP and fall in CBF. Physiological parameters (see below) were recorded from 20 minutes prior to SAH to 10 minutes or 3 hours after SAH. As animals regained consciousness and were able to breathe spontaneously they were returned to their cages and sacrificed at 10 minutes, 1, 3, 6 hours, or 24 hours after SAH.

Sham-operated animals were used as controls in this study. As described previously, sham surgery included all steps carried out in the surgery for SAH induction, except for internal carotid artery perforation [6]. Sham animals were matched in post-operative survival time to the SAH animals.

#### SAH Physiological Parameters

**Figure 1:**
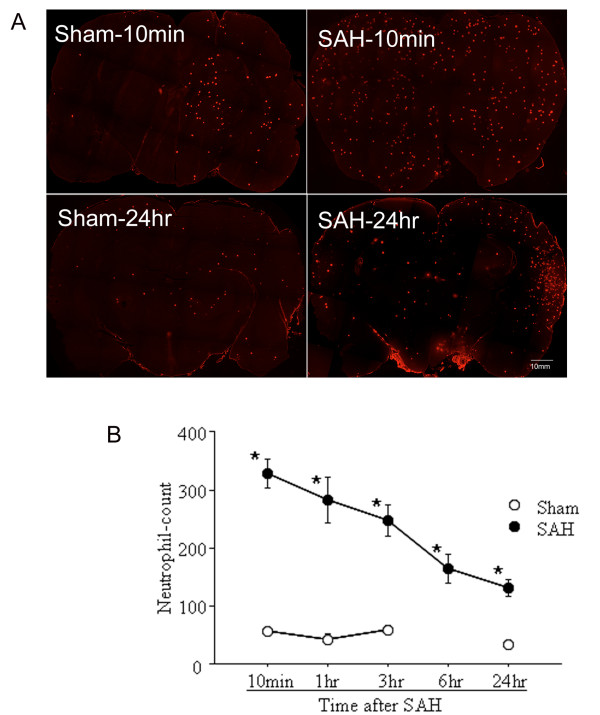
**Neutrophil infiltration after SAH**. A: Neutrophil staining in representative brain sections. Note that a large number of neutrophils are evident in the brain at 10 min and a smaller number at 24 hours after SAH. B: Number of neutrophils per whole coronal brain sections. Data are mean ± sem, N = 7 animals per time point. *p < 0.05.

Animals were assigned randomly to survival interval and treatment groups (N = 7 for SAH and 5 for sham surgery per time interval). ICP, CBF, and BP were recorded in real time. The average ICP rise at SAH from baseline was 5.4 ± 0.4 mmHg, with a peak of 60.0 ± 3.6 mmHg. CBF fell to 12.9 ± 1.4% of baseline at SAH and recovered to 47.7 ± 7.7% after 60 minutes. BP increased at SAH and returned to the baseline within five minutes. The ICP and CBF values indicated that rats experienced *moderate *SAH (Figure 1) [19]. The mortality 24 hours post SAH and sham surgeries in our laboratory on average are 29% and 10%, respectively.

#### Drug treatment

Three groups of animals were used. The first group was treated with vinblastine to deplete neutrophils (see table 1). This method of neutrophil depletion has frequently been used to study the role of neutrophil in cardiac, lung, traumatic brain and stroke injuries [20-23]. To deplete neutrophils, animals received vinblastine sulphate (cat. No: V1377, Sigma Aldrich MO, USA), along with Bicillin (cat. No: 3000979-A; King Pharmaceutical Inc. Bristol, TN, USA) and gentamicin (cat. No: G1272, Sigma Aldrich MO, USA) to prevent infection, 4 days before surgery (adapted from [21,22]). For vinblastine injection (N = 5 for SAH and 4 for Sham surgery) animals were anesthetized with ketamine-xylazine (80 mg/kg + 10 mg/kg; i.p), femoral venous catheters were inserted, and 0.5 mg/kg vinblastine sulphate, dissolved in saline, was administered intravenously. Bicillin (100 000 U) and gentamicin (10 mg/kg) were administered intramuscularly to prevent infection. Catheters were removed, and as the rats recovered from anaesthesia they were returned to their cages. All animals survived for 1 hour after SAH.

**Table 1:** Blood cell counts upon pharmacological treatments.

	anti PMN	anti PMN	Vinblastine	PDTC
Total WBC (10^3^/ul)	6.8	2	6	6
Neutrophils %	16	2	0.6	15
Platelets (10^3^/ul)	798	643	711	694

Animals were either untreated or were treated with vinblastine, anti PMN serum, or PDTC (see methods). Blood (200 ul) was drawn before SAH induction and analyzed for total white blood cells, neutrophil, and platelet counts using an LH-755 automated analyzer (Beckman Coulter, Brea, CA; n = 2 per treatment group). Shown are counts from a single animal. Normal rat white blood cell (WBC) counts are 6-18 10^3^/ul, neutrophils are 14-20%, and platelet counts are 500-1000 10^3^/ul [54].

The second group of animals was treated with rabbit anti rat PMN serum to deplete neutrophils (see table 1). Animals received daily intraperitoneal injection of 1 mL of saline-diluted (1:10) rabbit anti-rat PMN polyclonal antibody (cat. No: AIA51140, Accurate Chemical and Scientific NY, USA) for 3 days before SAH induction [24]. Controls for this group received daily IP injection of rabbit serum. The number of animals for anti PMN treatment is 6 and 2 for rabbit serum treatment. One anti PMN treated animal died within 1 hour after SAH.

The third group of animals was treated with pyrrolidine dithiocarbamate (PDTC) to reduce neutrophil activity (cat. No: P8765, Sigma Aldrich, MO, USA). The dose and the route of administration used were adapted from [25,26]. PDTC was dissolved in saline injected twice, 100 mg/kg i.p. at 12 hours and 50 mg/kg, one hour before surgery. The number of animals is 5 for immunostaining, 5 for permeability studies; see below. All animals survived for 1 hour after SAH.

### Histology

#### Brain preparation

Rats were perfused transcardially with saline and brains were rapidly removed, embedded in Tissue-Tek OCT compound (Miles, Elkhart, IN), and frozen in 2-methylbutane cooled in dry ice. 8 μm thick coronal brain sections were cut on a cryostat and thaw-mounted onto gelatin-coated slides. For neutrophil accumulation analysis 12 sections each 1 mm apart, from bregma +3.70 to - 8.7 mm [27] were used. For immunofluorescence, permeability, and zymography studies, sections located at bregma +0.2 and - 3.6 mm [27] were used.

#### Measurement of subarachnoid blood volume

The volume of blood surrounding the circle of Willis was estimated as described previously [18] by measuring blood areas in the interhemispheric region and basal subarachnoid space as seen in coronal brain sections (IPLab v3.0, Signal Analytics).

#### Microvascular permeability: FITC-albumin Extravasation

Animals were either untreated or PDTC treated and sacrificed 1 hour after SAH induction. Microvascular permeability was studied as previously reported [6]. Briefly, rats were sedated and the femoral artery was cannulated. FITC-albumin (Sigma, St. Louis, MO) was injected 15 minutes before sacrifice (bolus injection; 0.5 ml of 20 mg/ml preparation, N = 3 for untreated SAH control and 5 for PDTC treatment). Animals were killed by transcardiac perfusion with chilled saline followed by 1% chilled formaldehyde prepared freshly from paraformaldehyde (PFA). The brains were isolated and fixed in 1% PFA followed by solutions that contained 10%, 20% or 30% sucrose in 1% PFA. Fixation in each solution was carried out overnight at 4°C. Finally, the brains were embedded in Tissue-Tek OCT compound (Miles, Elkhart, IN), and frozen in 2-methylbutane cooled with dry ice and stored at -70°C until use.

### Immunofluorescence and Zymography

#### Reagents

1. Primary antibodies: goat monoclonal anti-collagen IV (Southern Biotechnology Associates Inc., Birmingham, AL; cat. no. 1340-01), rabbit polyclonal anti-collagen IV (Abcam, Inc, Cambridge, MA; cat. no AB6586), mouse monoclonal anti-rat endothelial cell antigen (RECA-1; MCA970R; Serotec Inc., Raleigh, NC; cat. no. MCA970R), mouse anti-neutrophil elastase (Senta Cruz Biotech, Santa Cruz, CA; cat. no.sc-55549) and rabbit polyclonal anti-neutrophil serum HB-199 (gift from Dr. D. Anthony, Oxford UK[28]). 2. Secondary antibodies: species-specific donkey anti-goat Alexa 350 (Invitrogen Corp. Carlsbad, CA; cat. no. A-21081), donkey anti-mouse Alexa 488 (Invitrogen Corp. cat. no. A-21202), and donkey anti-rabbit Rhodamine Red X (Jackson Immuno. Research; West Grove, PA; cat. no. 711-295-152). 3. DQ-gelatin solution (EnzCheck collagenase kit, Molecular Probes, Eugene, OR, USA; cat. no. E-12055).

#### Immunofluorescence

8 μm frozen brain sections were thawed and fixed for 15 minutes in 4% PFA. Sections were washed in physiological salt solution (PBS), and blocked in a solution of 3% normal donkey serum in PBS (DB). The sections were then incubated overnight at 4°C in a combination of anti-collagen IV, anti-RECA-1 and HB-199 or in a combination of anti-collagen IV and anti-neutrophil elastase (1:200 in DB) antibodies. Sections were washed and then incubated overnight at 4°C with species-specific secondary antibodies. Finally, sections were washed with PBS and coverslipped. Neutrophil elastase staining confirmed the specificity of HB-199 for neutrophils.

#### *In Situ *zymography and Immunofluorescence combination

8 μm frozen brain sections from untreated, vinblastine treated, anti PMN treated or PDTC treated animals sacrificed 1 hour after surgery were used (N = 5 per group). Unfixed brains were thawed and coated with a thin layer of FITC-labeled DQ-gelatin solution [3] containing collagen IV antibodies. The coated sections were incubated overnight at 37°C in a humid chamber, and then incubated overnight at 4°C with species-specific secondary antibodies. Finally, sections were fixed with chilled 4% PFA and coverslipped.

#### Immunostaining of FITC-albumin injected brains

8 μm frozen brain sections from untreated or PDTC treated animals sacrificed 1 hour after surgery were used (N = 5 per group). Sections were thawed and fixed in 4% PFA for 15 minutes. Sections were washed in PBS, and blocked in a solution of 5% normal donkey serum in PBS. The sections were then incubated overnight at with either rabbit anti-collagen IV, washed in PBS, incubated overnight at 4°C with donkey anti-rabbit Rhodamine Red-X, washed in PBS, and coverslipped with Vectashield (Vector labs, Burlingame, CA, USA).

### Data Acquisition

#### Physiology

CBF, ICP, and mean arterial blood pressure (MAP) were continuously recorded starting 20 minutes before SAH and ending 10 minutes, 1 hour, or 3 hour after SAH (PolyView software; Grass Instruments; MS, USA). CBF data were normalized to the baseline value averaged over 20 minutes prior to SAH, and subsequent values were expressed as a percentage of baseline [29].

#### Morphometry

All evaluations were performed by an observer blinded to specimen identity. Vessels studied were 100 μm or less in diameter and included pre- and post capillary arteries and venules. No distinction between capillaries and venules was made. Quantitative analysis was performed by manual counting or with IPLab (IPLab software v 3.63; Scanalytic Inc.; USA).

#### Neutrophil count

Composite montage images of whole coronal brain sections were acquired with a Leica DM-600 microscope (5 × objective, NA: 0.15) equipped with automated stage and montage acquisition software and assembled using MetaMorph (Molecular Devices, CA, USA). The number of neutrophils per section (both hemispheres, all brain regions) was manually counted in the whole section images.

#### Collagen IV and RECA-1 positive profile area fraction

10-12 fields per brain section were selected at random and analyzed for the number and area fraction of collagen IV and RECA-1 positive profiles and their colocalization. Stained profiles were isolated by intensity threshold segmentation with particle size gating. The IP lab was used to compute the area fraction as the summed area of segmented profiles in a field divided by the total area of the field.

#### Neutrophil-collagen IV or RECA-1 colocalization and parenchymal extravasation

HB-199 positive neutrophils were selected via threshold segmentation and gating. Collagen IV and RECA-1 positive profiles were selected as above. The total number of each labelled profile and the number of collagen IV and RECA-1 profiles that colocalized with neutrophil was determined using IP lab. Parenchymal extravasation of neutrophils was calculated by subtracting the number of collagen IV and HB-199 colocalized profiles from the total HB-199 image count.

#### *In situ *zymography-immunofluorescence combination

Four brain regions (basal, frontal and convexity cerebral cortex as well as caudoputamen), separated into right and left hemispheres, were analyzed by fluorescence microscopy (Axiophot; Carl Zeiss, USA). For quantitative analysis fluorescence images (2-3 fields per region and hemisphere) were recorded under constant illumination and exposure settings using a 20× objective (field area = 8 × 10^4 ^μm^2^), and were then studied for the number of collagen IV profiles positive for collagenase activity.

#### FITC-albumin extravasation

Collagen IV immunostaining was used to differentiate between vascular and parenchymal FITC-albumin deposits. Confocal images Z stacks were generated (see above). The number and area fraction of vascular and parenchymal FITC-albumin deposits in micrographs from basal, frontal and convexity cortex as well as in caudoputamen was determined using IP lab.

### Statistical analysis

All data points are presented as average ± standard error of mean (SEM). Each parameter (ICP, CBF, number and area fraction of collagen IV, RECA 1 or neutrophil immunostaining, zymography, and permeability data) was analyzed by two-way ANOVA (StatView v. 5.0.1, SAS Institute Inc. USA) with time and treatment query (control, SAH). Pairwise comparison used Fisher's PLSD *post-hoc *tests.

## Results

### Histology

#### Neutrophil infiltration

A large number of HB-199 stained neutrophils accumulated in brain as early as 10 min after SAH (Figure 1). Many of these neutrophils adhered to the endothelium of parenchymal vessels while others had entered the brain parenchyma. In addition, a small number of neutrophils were scattered within the blood which had accumulated in the subarachnoid space at the base of brain. The neutrophil count remained elevated at 1 hour and thereafter decreased with time (Figure 1B). In comparison to SAH animals, neutrophil numbers remained low in sham operated cohorts throughout the interval studied (P < 0.05).

Rostro-caudal differences in neutrophil invasion were assessed by counting HB-199 positive neutrophils in 12 brain sections each located 1 mm apart, using animals sacrificed 10 minutes after SAH. The results showed no significant rostro-caudal gradient in neutrophil numbers, confirming the global nature of ischemic injury after SAH (Table 2). Similarly, neutrophil number in different brain regions (basal, frontal and convexity cortex and caudoputamen) and between the two hemispheres was compared. The only significant regional difference was a decreased number of infiltrating neutrophils in the basal cortex. There were also significant interhemispheric differences in neutrophil count, with a larger count in the ipsilateral hemisphere (Table 3). This difference was present at 10 min, 3 hour, and 24 hours, while a trend towards significance was found at 1 hour (p = 0.07) and no significant difference was found at 6 hours (p = 0.31) after SAH. Interhemispheric difference in neutrophil count was also present in sham operated animals sacrificed at 10 minutes after the surgery but not thereafter. Neutrophils were not confined to vessels and in many cases had entered into the brain parenchyma near collagen IV stained vessels (see below). This brain parenchyma neutrophil infiltration was present at all examined time intervals after SAH. The number of parenchymal neutrophils after SAH was constant at approximately 40% of total neutrophils at all times (data not shown).

**Table 2:** Rostro-caudal differences in neutrophil infiltration 10 min after SAH.

Effect	Degrees of Freedom	F	p
Section	11	1.295	0.240
Brain region	1	12.02	0.0007
Section × Brain region	11	0.509	0.892

12 brain sections each located 1 mm apart, from bregma +3.70 to - 8.7 mm [27] were used. No significant difference in the neutrophil numbers among these brain sections was found. Data are mean ± sem, N = 5 animals

**Table 3:** Hemispheric and regional differences in neutrophil infiltration 10 min after SAH.

Effect	Degrees of Freedom	F	p
Hemisphere	1	7.868	0.0054
Brain area	3	6.406	0.0003
Hemisphere × Brain area	3	0.264	0.8512

Four brain regions (basal, frontal and convexity cortex and caudoputamen) were examined. A significant hemispheric and regional difference in neutrophil infiltration was found (ANOVA). Moreover no interaction between the hemispheres and brain regions was present, indicating global nature of ischemic brain injury after SAH. Similar hemispheric and regional differences in neutrophil count were present at 3, 6 and 24 hours after SAH (data not shown; see text for explanation). Data are mean ± sem, N = 5 animals.

#### Colocalization of neutrophil, collagen IV and RECA-1 immunostaining

**Figure 2:**
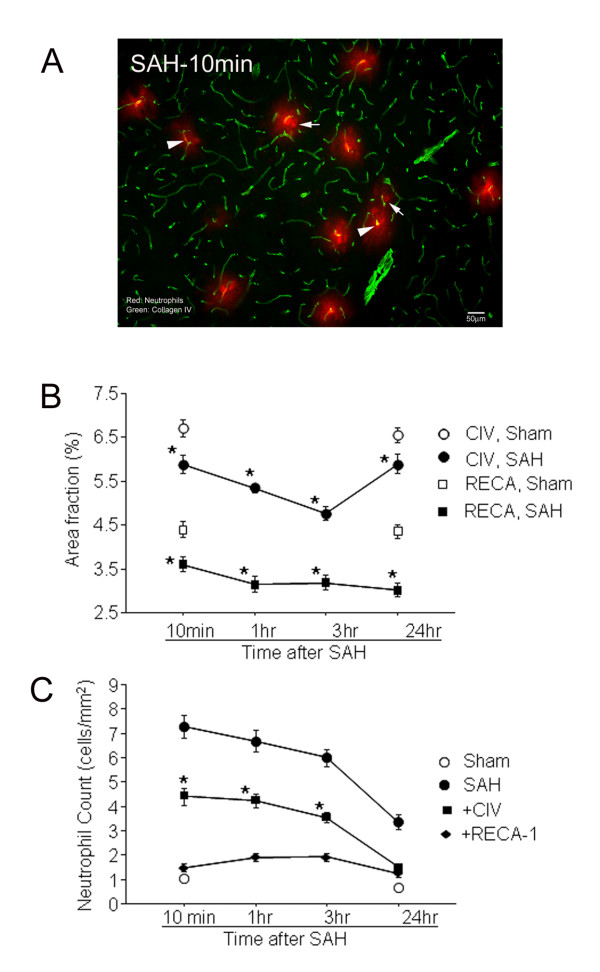
**Neutrophils in microvascular injury after SAH**. A: Representative image showing neutrophils in a brain section from an animal sacrificed 10 min after SAH. Note that some neutrophils (red) are within the collagen IV stained vessels (green; arrow heads) and others are present in the brain parenchyma (arrows). B: Area fractions of collagen IV and RECA-1 immunostaining in SAH and sham animals. Note that the area fraction of collagen IV and RECA-1 staining in SAH animals remained lower than the sham operated animals at all times examined. C: Numbers of neutrophils which are colocalized with collagen IV and RECA-1 after SAH. Filled circles: all neutrophils; filled squares: neutrophils that colocalized with collagen IV only; filled triangles: neutrophils that colocalized RECA-1 only. Open circles show all neutrophils in sham operated animals. Note that a greater number of neutrophils colocalized with collagen IV than with RECA-1 during the first 3 hours after SAH. Data are mean ± sem, N = 5 animals per time point and represent totals per whole coronal brain section * significantly different from sham operated animals (B) or from RECA-1 (C) at p < 0.05.

Animals were sacrificed at 10 minutes, 1 hour, 3 hour, or 24 hours after SAH. RECA-1 stained the endothelium and collagen IV stained the basal lamina of parenchymal vessels. Both vascular stains were reduced after SAH. RECA-1 staining was absent from most vascular sites that contained neutrophil (HB-199) staining (Figure 2A). Collagen IV staining was present in many but not all neutrophil positive vascular sites. This trend was observed at all time intervals in SAH animals but not in sham cohorts. Quantitative analysis showed that the area fractions of RECA-1 and collagen IV immunostaining were decreased at 10 minutes after SAH and remained decreased for 24 hours (Figure 2B). Qualitative examination of specimens revealed that, at any given time, more neutrophils colocalized with collagen IV than with RECA-1 (Figure 2C).

##### Drug treatment

The above studies find that a substantial rise in vascular and parenchymal neutrophils, as well as loss of RECA-1 and collagen IV immunostaining are present at 1 hour after SAH. Hence, in the drug study, the effect of reduction of neutrophil activity on microvascular injury was evaluated at 1 hour after SAH.

#### Physiological Parameters

**Figure 3:**
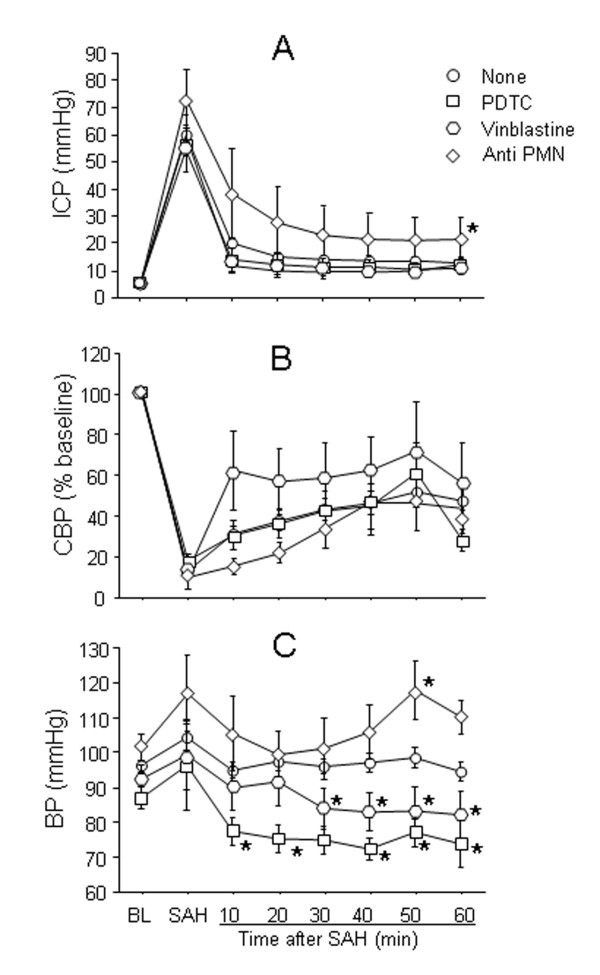
**Early physiological changes after SAH: Animals were either untreated or were treated with vinblastine, anti PMN serum, or PDTC**. ICP, CBF and BP were measured in real time from 20 minutes prior and 60 minutes post SAH (see methods). In A: note that ICP peak is similar in all groups but ICP decline in anti PMN group is significantly higher (25 ± 8 mmHg) than the untreated SAH animals (13 ± 1 mmHg). In B note that CAF fall and 60 minutes recovery is similar among animal groups. In C note that baseline BF and the transient increase in BP at SAH was similar among groups. There after BP decreased to lower levels in vinblastine and PDTC treated but not in anti PMN treated animals. Data are mean ± sem, N is 5-7 animals per treatment group. * significantly different at p < 0.05 from time matched untreated SAH animals.

ICP peak following hemorrhage was higher in anti PMN treated animals (77 ± 10 mmHg) than the rest of the treated or untreated animals (65.5 ± 5.2 mmHg) but did not reach significance (F = 0.9, p = 0.4; Figure 3). The decline and 60 minute plateau of ICP, however, was significantly higher in anti PMN treated animals as compared to untreated and vinblastine or PDTC treated animals (Controls: 13 ±1, PDTC: 25 ±8 mmHg; P = 0.05). This data suggests that although initial bleed at artery rupture was similar across treatment groups, bleeding continued for a longer duration in anti PMN animals (see blood quantitation). CBF fall at SAH (13.4 ± 1.1%) and 60 minute recovery (46 ± 6% of baseline) was similar in all animals groups (F = 1.4, p = 0.2).

#### Subarachnoid blood volume

**Figure 4:**
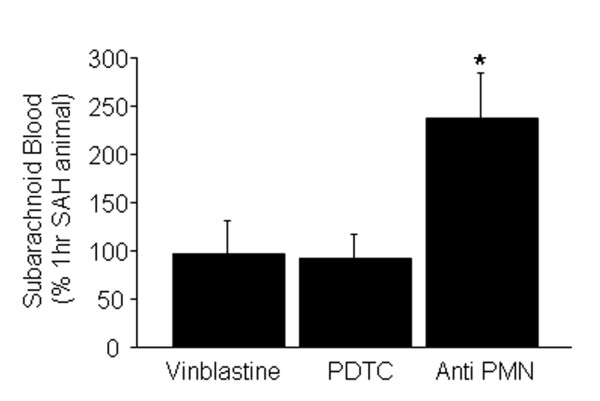
**Subarachnoid blood volume**. Animals were either untreated or were treated with vinblastine, anti PMN serum, or PDTC and sacrificed one hour after SAH induction. The volume of blood surrounding circle of Willis was measured (see methods). Subarachnoid hemorrhage blood volume in anti PMN but not vinblastine and PDTC treated animals was significantly greater than the untreated SAH animals. Data are mean ± sem, N is 5 animals per treatment group. * significantly different at p < 0.05 from time matched untreated SAH animals.

The volume of extravasated subarachnoid blood is another indicator of SAH intensity. We measured the volume of blood after SAH to determine if anti PMN treatment created a greater bleed. Quantitative analysis showed 2.5 times more subarachnoid blood in anti PMN treated animals as compared to untreated controls (P = 0.05, Figure 4). No difference in the subarachnoid blood volume among untreated and vinblastine or PDTC treated animals was found (P > 0.05; Figure 4).

#### Neutrophil immunostaining

**Figure 5:**
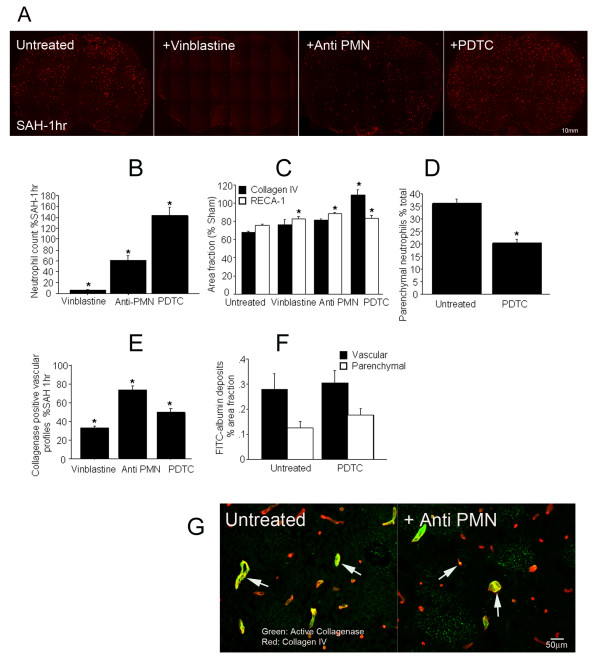
**Pharmacological reduction of Neutrophils and their activity**. A: Neutrophil staining in representative brain sections from untreated or vinblastine, anti PMN or PDTC treated animals sacrificed 1 hour after SAH. Note that fewer neutrophils are present in vinblastine and anti PMN treated animals and a large number are present in PDTC treated brains. B: Number of neutrophils in whole coronal brain sections. Values are % of untreated SAH animals. Neutrophils are decreased by vinblastin and anti-PMN treated and increased by PDTC treatment. C: RECA-1 (filled bars) and collagen IV (open bars) immunostaining following SAH. Values are area fractions in SAH animals as % of area fractions in sham-operated animals; both paramaters show trend or significant improvements in treated animals. D: Effect of PDTC treatment on the number of extravasated (parenchymal) neutrophils in SAH animals. Neutrophil extravasation is reduced by PDTC. E: Number of collagenase-positive profiles in treated SAH animals, given as % of values in untreated SAH animals. All three treatments reduce the extent of vascular collagenase activity. F: Effect of PDTC treatment on post-SAH intravascular tracer leakage. Values are area fractions of intravascular (closed bars) and extravascular (open bars) FITC-albumin deposits. G: Representative images of striatum showing vascular collagenase activity in untreated and anti PMN treated animals sacrificed at 1 hour after SAH. Arrows: collagen IV stained vessels (red) positive for collagenase activity (green). Data are mean ± sem. N = 5 animals per treatment group. * Significantly different at p < 0.05 from untreated SAH animals.

Animals were sacrificed 1 hour after SAH and brain sections were studied for neutrophil numbers. Neutrophil (HB-199) immunostaining revealed only a few neutrophils in the vinblastine treated specimens and a large number in the PDTC treated brains (Figure 5A). Quantitative analysis showed that vinblastine treatment reduced neutrophil count to less than 6%, and anti PMN treatment to approximately 60% of the untreated SAH animals (Figure 5B). In contrast, PDTC treatment *increased *neutrophil count by 14% compared to the untreated SAH animals (Figure 5B).

#### Neutrophil, collagen IV and RECA-1immunostaining

Animals were sacrificed 1 hour after SAH or sham surgery and the area fractions of collagen IV and RECA-1 positive profiles of treated animals was compared to untreated SAH and sham operated controls. Since vinblastine treatment itself reduces collagen IV immunostaining (data not shown), vinblastine treated shams were used as controls for that group. After SAH, significant reductions in the area fraction of collagen IV and RECA-1 positive profiles occurred in vinblastine-treated SAH animals as compared to vinblastine-treated shams (Figure 5C, p < 0.05). The SAH-induced reduction in collagen IV area fraction is significantly less in vinblastine treated SAH animals than in untreated SAH animals (untreated: 25% reduction, treated: 18% reduction; p = 0.02). A similar amelioration in RECA-1 loss after SAH was also observed, with marginal significance (untreated SAH 33% reduction, treated SAH 24% reduction; p = 0.09) (Figure 5C).

##### Anti PMN treatment

Rabbit serum treated animals, used as controls had similar reductions in RECA-1 and collagen IV staining as untreated SAH animals (P > 0.05). Consequently, untreated animals were used to compare the effect of anti PMN on RECA-1 and collagen IV staining. As in untreated and vinblastine treated animals, RECA-1 and collagen IV staining decreased following SAH in animals treated with the anti PMN serum. The extent of the reductions in RECA-1 and collagen IV staining, however, was significantly less in anti PMN compared to untreated animals (Figure 5C. RECA-1: 12% reduction [treated] vs 25% [untreated]; collagen IV: 18% reduction [treated] vs 33% [untreated]; P = 0.001, P = 0.003 respectively).

##### PDTC treatment

In contrast to vinblastine, anti PMN and untreated SAH animals, RECA-1 staining was preserved in PDTC treated animals: the majority of vascular profiles that were positive for neutrophils had retained endothelium staining. Quantitative analysis showed a significantly greater area fraction of RECA-1 positive profiles as compared to untreated shams and untreated SAH animals (108%) and a small but significant decrease (17%) in the area fraction of collagen IV positive vascular profiles in PDTC treated animals (Figure 5C, p < 0.05). Moreover, whereas in untreated animals over 35% of overall brain neutrophils had entered the parenchyma 1 hour after SAH, in PDTC treated animals this number was reduced to 20% (Figure 5D).

#### In situ zymography and collagen IV immunofluorescence

Untreated animals, as well as animals treated with vinblastine, anti PMN, or PDTC were sacrificed 1 hour after SAH. A large number of collagen IV immunostained vascular profiles that were positive for active collagenase were observed in zymograms of untreated animals. In comparison, fewer collagenase containing collagen IV profiles could be seen in treated animals (Figure 5G). The number of collagen IV immunostained profiles that were positive for collagenase activity was determined (Figure 5E). In untreated animals, 48% of collagen IV positive vessels had collagenase activity. Vinblastine, anti PMN and PDTC treatments reduced this number to 15% (75% reduction), to 72% (28% reduction) and 23% (68% reduction), respectively (Figure 5E, p = 0.0001).

*Microvascular Permeability *was assessed using intravascular albumin-FITC. This study was performed in PDTC pretreated animals, which showed the largest sparing of RECA-1 immunostaining following SAH. FITC-albumin deposits were numerous in brains of animals sacrificed 1 h after SAH. These deposits were scattered in both hemispheres and all brain regions (frontal, basal and convexity cortex as well as caudoputamen). Collagen IV staining distinguished between vascular (may indicate albumin incorporation in the growing platelet clot) and parenchymal (indicate extravasation) FITC-albumin deposits. In untreated animals, significantly more (p = 0.03) FITC-albumin deposits were present in the vessels (69% of total deposits) as compared to brain parenchyma (31% of total deposits). PDTC treatment did not affect the amount or distribution of FITC-albumin deposits (Figure 5F).

## Discussion

The present study investigated if pharmacological reduction of neutrophil activity reduces microvascular injury after SAH. The results demonstrate that depleting neutrophils or decreasing their activity prevents the loss of endothelium and collagen IV, and decreases collagenase activity after SAH.

### Neutrophil infiltration after SAH

Although animal and clinical studies indicate that a marked infiltration of neutrophil occurs 1-3 days after SAH [13-15], it has been unclear how soon after the initial bleed this process begins. Furthermore, most studies examined neutrophil accumulation in the subarachnoid space (animal studies) or in CSF (human studies) and did not provide information on neutrophils in brain microvasculature or parenchyma. Hence, we began this study by establishing the temporal profile of neutrophil accumulation in cerebral microvessels and in the brain parenchyma during the first 24 hours after SAH. Triple immunostaining for collagen IV, endothelium (RECA-1), and neutrophils (HB-199) allowed differentiation between vascular and parenchymal neutrophils. Moreover, saline perfusion at the time of animal sacrifice ensured that neutrophils floating in blood were removed and only those adhering to the vessel wall were counted as vascular neutrophils. This strategy revealed a massive time dependent accumulation of neutrophils in cerebral vessels and in brain parenchyma after SAH. As early as 10 minutes after SAH, a large number of neutrophils adhered to the vascular endothelium and had begun to infiltrate the brain parenchyma. The specific stimulus leading to neutrophil activation after SAH is still to be determined, but it is likely that platelet-derived cytokines play a role. A growing body of evidence establishes interplay between platelets and neutrophils in which activation of one promotes activation of the other [30-32]. Incidentally, it is important to note that platelets are activated within 10 minutes after SAH [8], and their interaction with vascular leukocytes is observed 2 hours later [33].

The increase in neutrophils 3 days after SAH, as observed in previous studies, may indicate that the neutrophil infiltration observed in this study persists for an extended period of time. This later phase of neutrophil infiltration is implicated in the development of delayed vasospasm [15,34]. The present study finds that the early phase of neutrophil infiltration is associated with early microvascular injury after SAH.

### Neutrophils and microvascular injury after SAH

If, when, and to what extent neutrophils contribute to early microvascular injury after SAH is not determined. An interaction of neutrophils with the vascular endothelium is essential in their recruitment to the injured area. The vascular consequences of this interaction include opening of interendothelial cell junctions and increased permeability, which facilitates neutrophil migration to the point of injury. Under pathological conditions, uncontrolled adhesion of neutrophils to the vascular endothelium occurs and results in acute endothelial injury [11,12]. In addition, vascular neutrophils plug and obstruct the vessel lumen to limit flow, thus exacerbating brain injury and creating local ischemia [35].

In the present study immunostaining of RECA-1 decreased after SAH, indicating damage to the vascular endothelium. Of note, RECA-1 was often missing from vascular sites that contained neutrophils. Furthermore, with time collagen IV also disappeared from most RECA-1 deficient sites. This finding implies a contribution of neutrophils in endothelial and collagen IV loss after SAH. A similar combination of vascular neutrophil accumulation, blood-brain barrier destruction, and collagen IV degradation is observed upon hemorrhagic transformation in humans and animals receiving tissue plasminogen activator following occlusive ischemic stroke, but this result develops over 24 hours [36,37]. The presence of these phenomena at 10 minutes in our studies indicates that the nature of vascular injury after SAH and ischemic stroke may be similar, but that injury develops at a much faster pace after SAH as compared to occlusive ischemic stroke.

Early alteration in the structure and function of cerebral vasculature is documented after SAH. This includes loss of endothelial antigens, detachment of endothelium from the basal lamina, degradation of collagen IV, increase in permeability and decrease in perfusion [1-6]. It is interesting to note that all of these events have a similar temporal profile as the appearance of vascular and parenchymal neutrophils; all are present at 10 minutes and persist for at least 24 hours after SAH. This implies a role for neutrophils in early vascular injury after SAH.

Neutrophils can cause and promote vascular injury by a number of mechanisms: (1) they can injure endothelium by reactive oxidant species (such as hydrogen peroxide and superoxide) released during respiratory burst, and by elastases and proteases released during degranulation [12,38,39]. (2) They can degrade basal lamina by releasing proteolytic enzymes, including collagenase and MMP-9, which are known to digest collagen IV [36,37]. (3) The neutrophil-derived enzyme myeloperoxidase can catalytically consume nitric oxide (NO) as a substrate, which promotes endothelial dysfunction and constriction [40,41]. An early decrease in cerebral NO, endothelial dysfunction, and constriction is established after SAH [4,42,43]. Incidentally, decrease in cerebral NO occurs around 10 min after the initial bleed, just as the number of vascular neutrophils reaches its peak. A role of myeloperoxidase in NO depletion remains to be evaluated.

### The effect of depleting or limiting neutrophil activity on early microvascular injury after SAH

Most strategies for decreasing the activity of neutrophils are aimed towards reducing their vascular accumulation or activation [25,44,45]. We examined if neutrophil depletion by vinblastine and anti PMN serum, or reducing neutrophil induced oxidative stress by PDTC, could prevent vascular injury after SAH. We found that neutrophil depletion reduces vascular collagenase activation and protects against loss of collagen IV and endothelium after SAH. However, side effects associated with vinblastine and anti PMN treatments make them unsuitable as therapies. Anti PMN creates long lasting bleeds from the ruptured artery, generating a larger hemorrhage. As the platelet count remains unchanged by anti PMN, the long lasting bleeds may indicate a disturbance in the coagulation pathway, delaying clot formation at the site of arterial rupture. Vinblastine, on the other hand significantly weakens the vascular cytoskeleton. This side effect, resulting from disruption of microtubules and inhibition of collagen synthesis and secretion, is well documented [46].

PDTC, the third pharmacological agent examined in this study, is an antioxidant and an inhibitor of transcription factor nuclear factor kappa B (NF-κB). As an antioxidant, PDTC scavenges neutrophil-derived oxidants, especially hypochlorous acid (HOCl). HOCl inactivates plasma proteinase inhibitors and thereby prolongs neutrophil elastase activity; in addition, it activates neutrophil-derived collagenase and gelatinase (MMP-9). Together, these enzymes promote the degradation of the extracellular matrix [47,48]. Thus, by scavenging HOCl, PDTC limits elastase and collagenase activity, and decreases the deleterious effects they have on vascular tissue. NF-κB activation is a central event in the basal and inducible expression of various inflammatory cytokines in human neutrophils [49]. Hence, PDTC represents a double edge sword that could prevent or reduce the entire chain of inflammatory events induced by neutrophils. Indeed, PDTC treatment has been used to reduce ischemia/reperfusion injury and infarct size after experimental stroke [25,26].

In the present study PDTC treatment significantly increased the number of vascular neutrophils while reducing the number that escaped into the parenchyma. Recently, Langereis et al., have found that inhibition of NF-κB activation in neutrophils increases their survival [50]. Increased vascular neutrophil accumulation in PDTC treated animals may indicate an inhibition of NF-κB activation in neutrophils. NF-κB inhibition may also be the mechanism underlying the protective effects PDTC exerts on post SAH vascular collagen IV and RECA-1 immunostaining and on the reduction in post SAH vascular collagenase we find following PCTD treatment. Another known effect of PDTC, not related to neutrophil activity, is the inhibition of endothelial cell apoptosis [51]. This effect occurs 24 hours after SAH [52]; it is likely not involved in the phenomena we describe here at 1 hour after SAH.

That cerebral microvessels are only partially spared by the treatments tested here most likely reflects the contribution of elements other than neutrophils to microvascular damage following SAH. Activated platelets and alterations in the nitric oxide pathway represent two other important aspects of this complex and multifaceted process [5,42,53].

## Conclusions

In *conclusion*, we have found that pharmacological reduction of the activity of neutrophils reduces microvascular injury after SAH. This finding suggests that neutrophil-targeted interventions may prove beneficial in ameliorating brain injury after SAH.

## Competing interests

The authors declare that they have no competing interests.

## Authors' contributions

RF, AM and WB carried out animal studies and immunostaining and were responsible for data collection. EP participated in blood cell analysis and neutrophil depletion protocols. VF participated in the study design, data analysis and interpretation and in the writing of the manuscript. FAS conceived the study and the design, coordinated the work and the writing of the manuscript. All authors have approved the final manuscript.
